# Antibody-Dependent Immune Complex Signaling and Inflammatory Cytokine Responses in Acute Febrile Illness: A Mechanistic Study in Arboviral and Leptospiral Infection

**DOI:** 10.1093/ofid/ofag300

**Published:** 2026-05-14

**Authors:** Peter Mac Asaga, Vijeesh Kadukkatti, Philomena Airiohuodion, Brijil Mathew, Sunday Omilabu

**Affiliations:** Institute for Infection Prevention and Control, Medical Center, University of Freiburg, Breisacher str 115b, 79106 Freiburg, Germany; RWTH Uniklinik, Aachen, Institute of Occupational Health, Social and Enviromental Medicine, Pauwelsstr 30, 52074 Aachen, Germany; Special Programme for Research and Training in Tropical Diseases, World Health Organization, Geneva, Switzerland; Aster MIMS Hospital, Kannur 670621, Kerala, India; CHAHZY Central Research Laboratory, College of Medicine, University of Lagos, Lagos, Nigeria

**Keywords:** antibody-dependent enhancement, arbovirus, cytokine, Fc receptor, immune complex

## Abstract

**Background:**

Arboviral infections are characterized by heterogeneity in inflammatory responses and clinical outcomes. While antibodies are essential for protection, antibody–antigen immune complexes may modulate innate immune signaling through Fc receptor engagement. The extent to which immune complex signaling contributes to coordinated cytokine responses in human arboviral infection remains incompletely defined.

**Methods:**

We conducted a mechanistic immunology study nested within a prospective cohort of patients with acute arboviral infection in southern India. Using paired ex vivo stimulation assays, we compared cytokine responses following antigen-only and antibody–antigen immune complex stimulation. Fc dependence was interrogated using Fcγ receptor blockade and immunoglobulin G (IgG) subclass depletion. Cytokines were quantified using the LEGENDplex Human Inflammation Panel (BioLegend). Principal component analysis was applied to identify coordinated cytokine response patterns, and multivariable regression models were used to assess associations with humoral immune state.

**Results:**

Among 80 participants, immune complex stimulation was associated with higher concentrations of pro-inflammatory cytokines compared with antigen alone. This difference was attenuated by Fcγ receptor blockade and was influenced by antibody subclass composition. Principal component analysis revealed a coordinated cytokine pattern characterized by positive contributions from interleukin-6 (IL-6), tumor necrosis factor, IL-1β, and CXCL10, with an inverse contribution from IL-10. In multivariable analysis, individuals with lower neutralization capacity or altered IgG subclass profiles exhibited higher inflammatory cytokine program scores (*β* = +1.80 standard deviation; 95% confidence interval 1.54–2.06; *P* = 7.18 × 10^−22^).

**Conclusions:**

These findings suggest that antibody-dependent immune complex signaling may contribute to inflammatory cytokine responses during acute arboviral and leptospiral infection.

Arboviral infections represent a growing global public health challenge, with increases in incidence, geographic spread, and clinical burden observed over the past decade. Dengue, chikungunya, and related arboviruses have expanded into new regions, driven by climate change, urbanization, and vector adaptation, leading to recurrent outbreaks that disproportionately affect low- and middle-income countries [1–3]. Despite advances in surveillance, vaccine development, and vector control, the mechanisms underlying disease heterogeneity and severe inflammatory manifestations remain incompletely understood.

A characteristic feature of severe arboviral disease is dysregulated inflammation, which may include elevated cytokine production, endothelial activation, and vascular permeability, often accompanied by thrombocytopenia and coagulopathy [4–6]. Previous studies have documented associations between individual cytokines, such as interleukin-6 (IL-6), tumor necrosis factor (TNF), and interferon-inducible chemokines, and adverse clinical outcomes [7–9]. However, the interpretation of these associations is complicated by the coordinated nature of cytokine responses and the challenge of distinguishing causal mechanisms from downstream inflammatory amplification. There is increasing recognition that disease pathogenesis may be better understood in terms of integrated cytokine programs rather than isolated mediators [10, 11].

Humoral immunity plays a central but complex role in arboviral infection. Neutralizing antibodies are essential for viral clearance and long-term protection, yet antibodies may also contribute to immunopathology under certain conditions [12, 13]. In dengue virus infection, secondary exposure to antigenically related viruses has been associated with antibody-dependent enhancement of infection and inflammation, a phenomenon that has been described at both epidemiological and experimental levels [14–16]. Beyond potential enhancement of viral entry, antibodies form immune complexes that engage Fcγ receptors on myeloid cells, which may trigger downstream inflammatory signaling pathways [17–19].

Fc receptor–mediated immune complex signaling represents a potential interface between humoral and innate immunity. Engagement of activating and inhibitory Fcγ receptors may integrate signals from antibodies, antigen burden, and innate pattern recognition receptors, leading to modulation of cytokine production [20–22]. Antibody characteristics, such as subclass distribution, Fc glycosylation, and affinity for Fcγ receptors, have been identified as potential determinants of these downstream responses [23–25]. System serology approaches have suggested that antibody quality, rather than titer alone, may predict inflammatory outcomes across a range of infectious and inflammatory diseases [26–28]. However, direct mechanistic evidence linking antibody-dependent immune complex signaling to coordinated cytokine programming in acute human infection remains limited.

In this study, we investigated antibody-dependent immune complex signaling as a potential determinant of inflammatory cytokine responses in human arboviral infection. Using paired antigen-only and immune complex stimulation assays, combined with Fcγ receptor blockade and immunoglobulin G (IgG) subclass depletion, we interrogated Fc-dependent cytokine induction in patient-derived samples. We further applied multivariable statistical modeling to identify coordinated cytokine response patterns and to assess how humoral immune state relates to these responses across individuals.

## METHODS

### Study Design and Participants

This study was conducted as a prospective observational cohort nested within an ongoing investigation of acute febrile illness in southern India. Consecutive patients presenting with suspected arboviral infection were enrolled following informed consent. Inclusion criteria comprised acute febrile illness (axillary temperature ≥38.0 °C) with symptom onset within 7 days. Individuals with known chronic inflammatory or autoimmune conditions, immunosuppressive therapy, or pregnancy were excluded. Clinical severity was classified using predefined criteria based on clinical signs, laboratory parameters, and need for hospitalization or intensive care, adapted from the World Health Organization dengue severity classification.

A mechanistic immunology subset was selected for immune complex and Fc perturbation experiments based on sample availability and completeness of serological data. This subset was not enriched for disease severity and was intended to be representative of the broader cohort in terms of age, sex, and infection category.

Samples were collected during a large regional outbreak of acute febrile illness with co-circulating dengue virus, chikungunya virus, and leptospira, allowing investigation of immune complex–driven inflammatory responses across a clinically relevant spectrum of infectious exposures.

### Sampling and Specimen Processing

Peripheral venous blood samples (10 mL) were collected at enrollment prior to therapeutic interventions using EDTA-coated tubes. Whole blood was processed within 2 hours of collection at the study site laboratory. Plasma was separated by centrifugation at 1500 *g* for 10 minutes at room temperature and aliquoted into cryogenic vials for cytokine assays, serological testing, and functional antibody assays. All samples were stored at −80 °C until analysis. Repeated freeze–thaw cycles were avoided.

### Pathogen Confirmation and Serology

Laboratory confirmation of dengue virus infection was performed using NS1 antigen detection and dengue IgM and IgG capture enzyme-linked immunosorbent assays, according to the manufacturer's instructions. Chikungunya virus infection was confirmed using an anti-chikungunya virus IgM enzyme-linked immunosorbent assay. Leptospirosis was confirmed using an IgM enzyme-linked immunosorbent assay or microscopic agglutination testing performed at the regional reference laboratory. Dengue virus RNA was detected by real-time reverse transcription polymerase chain reaction using a validated multiplex assay.

Neutralizing antibody titers against dengue virus serotypes 1–4 were determined using plaque reduction neutralization tests performed on Vero cells as previously described. The PRNT_50_ titer was calculated as the reciprocal of the highest serum dilution that reduced plaque counts by ≥50% compared with virus-only controls. A PRNT_50_ titer ≥40 was considered indicative of protective neutralizing antibody levels.

Immunoglobulin G subclass profiles (IgG1, IgG2, IgG3, and IgG4) against dengue virus envelope protein were quantified using a bead-based multiplex immunoassay. Results were expressed as median fluorescence intensity. The IgG3 ratio was calculated as IgG3 median fluorescence intensity divided by total IgG median fluorescence intensity.

Participants were classified into humoral immune groups based on PRNT_50_ titer and IgG3 ratio: high neutralization with IgG3-enriched profile (PRNT_50_ ≥ 40 and IgG3 ratio above the cohort median), low neutralization or altered IgG subclass profile (PRNT_50_ < 40 or IgG3 ratio below the cohort median), and IgG negative (dengue IgG negative by ELISA).

### Immune Complex Stimulation Assays

Whole-blood stimulation assays were performed using a standardized protocol adapted from previous studies. Heparinized whole blood was diluted 1:5 in RPMI 1640 medium supplemented with heat-inactivated fetal bovine serum, L-glutamine, and antibiotic–antimycotic solution in round-bottom tissue culture plates. Dengue virus envelope protein was used as a standardized immune complex stimulus to probe Fc-dependent inflammatory signaling in human whole blood, independent of the infecting pathogen. Leptospirosis cases were included as a nonarboviral comparator group to assess whether immune complex–driven inflammatory responses generalize beyond arboviral infections.

For antigen-only stimulation, diluted whole blood was incubated with purified recombinant dengue virus envelope protein or unstimulated (medium-only) control. For immune complex stimulation, dengue virus envelope protein was preincubated with autologous plasma (10% v/v) for 30 minutes at 37 °C to allow immune complex formation before addition to diluted whole blood. All conditions were performed in technical duplicate within a single experiment per participant. Repeat experiments on independent days were not performed owing to limited sample volume.

Plates were incubated for 24 hours at 37 °C with 5% CO_2_ in a humidified incubator. Following incubation, plates were centrifuged, and cell-free supernatants were harvested and stored at −80 °C until cytokine quantification.

### Fcγ Receptor Blockade and IgG Subclass Depletion

For Fcγ receptor blockade experiments, immune complex stimulation was performed in the presence of blocking antibodies against FcγRIIa and FcγRIIIa added simultaneously to achieve dual receptor blockade. Isotype-matched control antibodies were used at equivalent concentrations.

For IgG3 depletion experiments, autologous plasma was depleted of IgG3 using a 2-step immunoaffinity protocol. Total IgG was first captured using protein G affinity chromatography and eluted under acidic conditions, followed by immediate neutralization. Eluted IgG was subsequently incubated with anti-human IgG3 affinity beads. Immunoglobulin G3-depleted supernatant was collected by centrifugation and reconstituted to the original plasma IgG concentration using centrifugal filter devices. Depletion efficiency was confirmed by enzyme-linked immunosorbent assay, with >95% reduction in IgG3 levels achieved. For IgG3 add-back experiments, purified human IgG3 was added to IgG3-depleted plasma at physiological concentrations prior to immune complex formation.

### Phospho-SYK Measurement

To assess proximal FcγR signaling, phosphorylated SYK (pSYK) was measured in CD14^+^ monocytes by intracellular flow cytometry. Whole blood was stimulated with immune complexes under the conditions described above for 5 minutes at 37 °C, then immediately fixed with paraformaldehyde and permeabilized with methanol. Cells were stained with anti-CD14-APC and anti-pSYK(Y525/526)-PE antibodies. Median fluorescence intensity of pSYK was measured on a flow cytometer and normalized to the intact immune complex condition for each donor.

### Cytokine Quantification

Cytokine concentrations (IL-6, TNF, IL-1β, IL-10, and CXCL10/IP-10) were measured in culture supernatants using a bead-based multiplex immunoassay, according to the manufacturer's instructions. Briefly, thawed supernatants were incubated with capture beads in V-bottom plates, followed by incubation with biotinylated detection antibodies and streptavidin–phycoerythrin. Beads were acquired on a flow cytometer, and data were analyzed using the manufacturer's analysis software. Standard curves were generated using serially diluted cytokine standards, and concentrations were calculated using a 5-parameter logistic curve fit.

Cytokine concentrations below the lower limit of detection were assigned half the lower detection limit for statistical analysis. The interassay coefficient of variation was <15% for all analytes. All assays were performed blinded to clinical severity and humoral immune status.

C-reactive protein concentrations were measured in plasma using an enzyme-linked immunosorbent assay. Angiopoietin-2 concentrations were measured using a commercial immunoassay. Complete blood counts, including platelet counts, were performed on an automated hematology analyzer at the study-site clinical laboratory within 2 hours of sample collection.

### Statistical Analysis

All statistical analyses were performed using R software. Data visualization was performed using standard plotting packages. Cytokine concentrations were log_10_ transformed prior to analysis to account for right-skewed distributions. Paired comparisons between stimulation conditions were conducted using 2-sided Wilcoxon signed-rank tests. Unpaired comparisons between groups were performed using Mann–Whitney *U* tests.

Principal component analysis (PCA) was performed on log-transformed immune complex–stimulated cytokines (IL-6, TNF, IL-1β, IL-10, and CXCL10) with centering and scaling. The first principal component was interpreted as representing an inflammatory–regulatory cytokine axis and was used as a composite outcome in downstream analyses. Principal component analysis loadings and variance explained were extracted for reporting.

Multivariable linear regression models were used to examine associations between humoral immune state and cytokine program scores, adjusting for age, sex, and infection category. Model assumptions were assessed by inspection of residual plots. Effect sizes are reported with 95% confidence intervals (CIs). The false discovery rate correction was applied where multiple cytokines were tested. A 2-sided *P*-value of <.05 was considered statistically significant.

To assess whether viral load confounded the association between humoral immune state and cytokine programming, we performed secondary analyses restricted to participants with arboviral infection and detectable viremia (n = 53). Spearman correlation was used to assess the univariate relationship between log_10_ viremia and PC1 score. Multivariable linear regression models were then compared with and without viremia as a covariate.

### Sample Size

This was an exploratory mechanistic study, and formal sample size calculations were not performed a priori. The sample size of 80 participants for the mechanistic subset was determined by sample availability and was considered sufficient for exploratory analyses of immune complex–mediated cytokine responses based on previous studies of similar design [29, 30].

### Ethical Approval

The study protocol was reviewed and approved by the Institutional Ethics Committee of Indian Council of Medical Research (NO 372/2022). Written informed consent was obtained from all participants or their legally authorized representatives prior to enrollment. For participants aged 12–17 years, written assent was obtained in addition to parental/guardian consent. All study procedures were conducted in accordance with the Declaration of Helsinki (2013 revision) and the Indian Council of Medical Research National Ethical Guidelines for Biomedical and Health Research Involving Human Participants (2017).

## RESULTS

### Participant Characteristics

The mechanistic study cohort comprised 80 participants with acute arboviral or leptospiral infection (Table 1). The median age was 40 years (interquartile range [IQR] 31–49), and 41 participants (51.2%) were male. Infection categories were distributed as follows: dengue (n = 26), chikungunya (n = 27), and leptospirosis (n = 27). Disease severity was classified as mild in 37 participants, moderate in 23, and severe in 20.

**Table 1.** Baseline Characteristics of the Mechanistic Study Cohort (n = 80)

Characteristic	Value
Age, y, median (IQR)	40 (31–49)
Male sex, n (%)	41 (51.2)
Infection category, n	
Dengue	26
Chikungunya	27
Leptospirosis	27
Disease severity, n	
Mild	37
Moderate	23
Severe	20

Data are presented as median (interquartile range, IQR) or n (%).

### Immune Complex Stimulation is Associated With Higher Inflammatory Cytokine Concentrations

**Figure 1.**
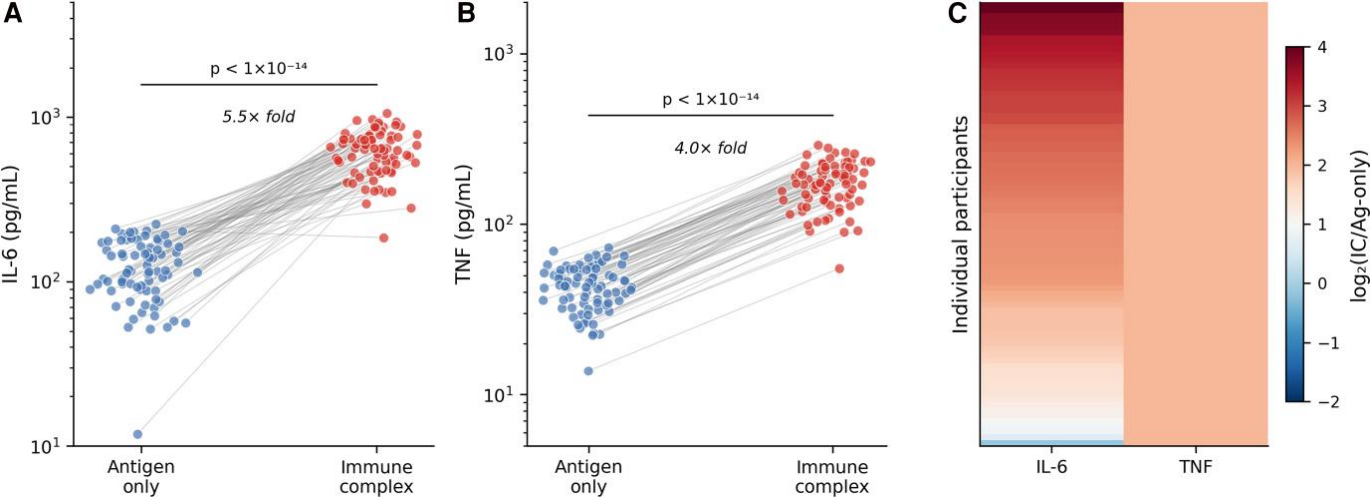
Immune complex stimulation amplifies inflammatory cytokine responses (n = 80). *A*, Paired interleukin-6 concentrations following antigen-only and immune complex stimulation. *B*, Paired tumor necrosis factor concentrations. *C*, Heat map of individual-level log_2_-fold change (immune complex/antigen-only). Lines connect paired observations from the same individual. *P* values from paired Wilcoxon signed-rank tests.

To investigate whether antibody–antigen immune complexes modulate inflammatory signaling, we compared cytokine responses following antigen-only stimulation and immune complex stimulation within the same individuals. Immune complex stimulation was associated with higher concentrations of pro-inflammatory cytokines compared with antigen alone (Table 2; Figure 1*A* and 1*B*). Unstimulated (medium-only) controls yielded cytokine concentrations at or below the lower limit of quantification for all analytes (Table 2), confirming that the observed responses were attributable to stimulation conditions rather than constitutive secretion.

**Table 2.** Immune-Complex–Dependent Cytokine Responses

Cytokine	Unstimulated Control Median (IQR)	Antigen-only Median (IQR)	Immune Complex Median (IQR)	*P V*alue
IL-6 (pg/mL)	≤LLOQ	117 (92–163)	644 (514–759)	<1 × 10^−14^
TNF (pg/mL)	≤LLOQ	44 (35–52)	175 (139–209)	<1 × 10^−14^
IL-1β (pg/mL)	≤LLOQ	…	201 (156–267)	…
IL-10 (pg/mL)	≤LLOQ	…	59 (41–84)	…
CXCL10 (pg/mL)	≤LLOQ	…	681 (512–894)	…

*P* values from paired Wilcoxon signed-rank test.

Abbreviations: IL, interleukin; IQR, interquartile range; ≤LLOQ, at or below the lower limit of quantification; cytokine concentrations in unstimulated (medium-only) controls were uniformly low and below or near the lower limit of quantification for all analytes; TNF, tumor necrosis factor; …, not measured in antigen-only condition.

Median IL-6 concentration was 117 pg/mL (IQR 92–163) following antigen-only stimulation compared with 644 pg/mL (IQR 514–759) following immune complex stimulation (paired Wilcoxon signed-rank test, *P* < 1 × 10^−14^). Similarly, median TNF concentration was 44 pg/mL (IQR 35–52) with antigen alone compared with 175 pg/mL (IQR 139–209) with immune complex stimulation (*P* < 1 × 10^−14^). This pattern was observed consistently across infection categories and was evident at the individual level (Figure 1*C*).

### Fc Perturbation Modulates Immune Complex–Induced Cytokine Responses

**Figure 2.**
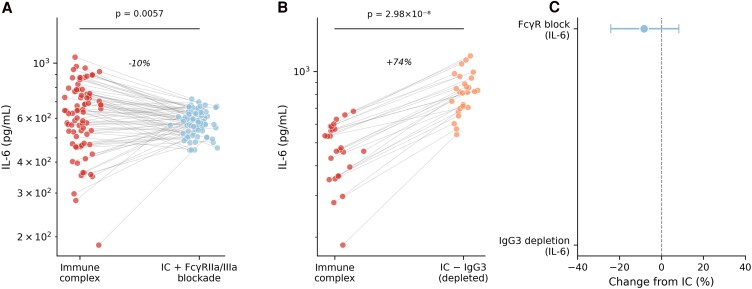
Fc perturbation modulates immune complex–induced cytokine responses. *A*, Interleukin-6 (IL-6) concentrations following dual FcγRIIa/IIIa blockade (n = 80). *B*, IL-6 concentrations following IgG3 depletion (n = 30). *C*, Summary of percentage change from intact immune complex condition. *P* values from paired Wilcoxon signed-rank tests.

To assess whether the observed cytokine differences were mediated through Fc-dependent mechanisms, we examined cytokine responses following Fcγ receptor blockade and IgG subclass depletion (Table 3; Figure 2).

**Table 3.** Fc Perturbation Effects on Immune-Complex Interleukin-6 (IL-6) Responses

Condition	Median IL-6 (pg/mL)	Paired *P* Value
Immune complex (intact serum)	644	…
Immune complex + FcγRIIa/IIIa blockade	577	.0057
Immune complex + IgG3 depletion	821	2.98 × 10^−8^

*P* values from paired Wilcoxon signed-rank test comparing each perturbation condition to unperturbed immune complex stimulation with intact serum.

Dual FcγRIIa/IIIa blockade was associated with lower immune complex–induced IL-6 responses compared with unblocked conditions. Median IL-6 concentration was 644 pg/mL with immune complex stimulation alone compared with 577 pg/mL following Fcγ receptor blockade (paired Wilcoxon test, *P* = .0057; Figure 2*A*). Immunoglobulin G3 depletion was associated with altered immune complex cytokine responses. Median IL-6 concentration increased to 821 pg/mL following IgG3 depletion compared with 644 pg/mL with intact serum (*P* = 2.98 × 10^−8^; Figure 2*B*).

### IgG3-Dependent Immune Complex Signaling is Mediated Through FcγRIIIa-SYK Activation

**Figure 3.**
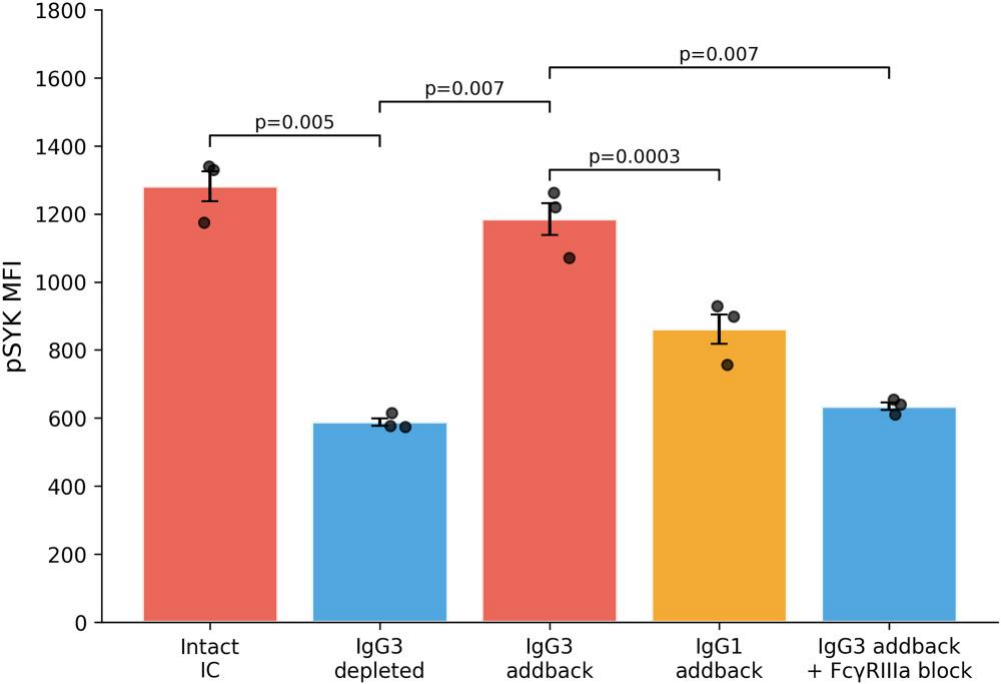
Phospho-SYK median fluorescence intensity in CD14^+^ monocytes following immune complex stimulation under IgG3 depletion, add-back, and FcγRIIIa blockade conditions (n = 3 donors). Bars represent mean ± standard error of the mean; individual data points shown. *P* values from paired t-tests.

To determine whether the observed cytokine differences reflected proximal FcγR signaling, we measured phosphorylated SYK (pSYK) in CD14^+^ monocytes following immune complex stimulation (Supplementary Table 1). Depletion of IgG3 reduced pSYK levels by 54% compared with intact immune complexes (*P* = .005). Adding back purified IgG3 restored pSYK to 93% of the level observed with intact immune complexes (*P* = .007 vs depleted), whereas IgG1 add-back achieved only partial restoration (67%; *P* = .0003 vs IgG3 add-back). Importantly, FcγRIIIa blockade abolished the IgG3-mediated pSYK response, reducing signaling to levels similar to those seen with IgG3 depletion (*P* = .007). Together, these findings support a mechanistic pathway in which IgG3-containing immune complexes engage FcγRIIIa, leading to SYK phosphorylation and downstream cytokine production (Supplementary Table 1; Figure 3).

### Immune Complex Stimulation Elicits Coordinated Cytokine Response Patterns

**Figure 4.**
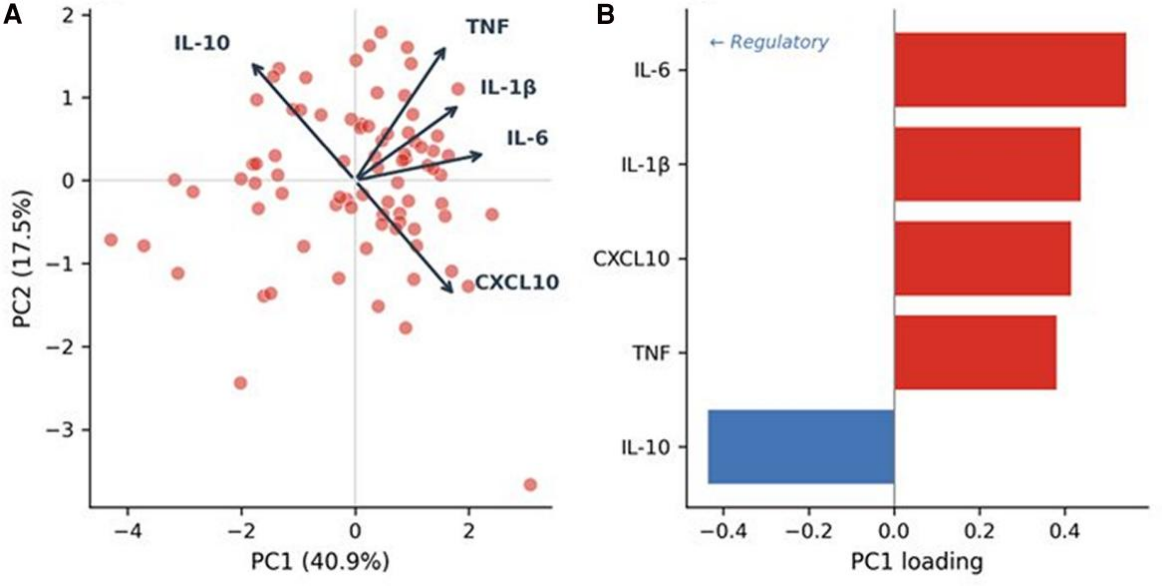
Principal component analysis (PCA) of immune complex cytokine responses (n = 80). *A*, PCA biplot showing individual scores and cytokine loading vectors on PC1 (40.9%) and PC2 (17.5%). *B*, PC1 loadings for each cytokine; interleukin-10 (blue) loads inversely, indicating counter-regulatory behavior.

Principal component analysis was performed on log-transformed immune complex–stimulated cytokine concentrations (IL-6, TNF, IL-1β, CXCL10, and IL-10). The first principal component (PC1) accounted for 40.9% of the total variance and represented a dominant inflammatory axis, with positive contributions (loadings) from IL-6 (0.545), IL-1β (0.439), CXCL10 (0.416), and TNF (0.382), and a negative contribution from IL-10 (−0.437) (Table 4; Figure 4*A* and 4*B*). The second principal component (PC2) explained an additional 17.5% of the variance (Supplementary Figure 1).

**Table 4.** Principal Component Analysis of Immune-Complex Cytokine Responses

Cytokine	PC1 Loading	PC2 Loading
IL-6	0.545	0.117
TNF	0.382	0.592
IL-1β	0.439	0.334
CXCL10	0.416	−0.502
IL-10	−0.437	0.521
Variance explained (%)	40.9	17.5
Cumulative variance (%)	40.9	58.4

Principal component analysis performed on log-transformed immune complex–stimulated cytokine concentrations. PC1 represents the inflammatory-regulatory cytokine program, with positive loadings indicating pro-inflammatory contribution and negative loadings indicating regulatory contribution.

Abbreviations: IL, interleukin; IQR, interquartile range; TNF, tumor necrosis factor.

### Humoral Immune State is Associated With Immune Complex Cytokine Program Scores

**Figure 5.**
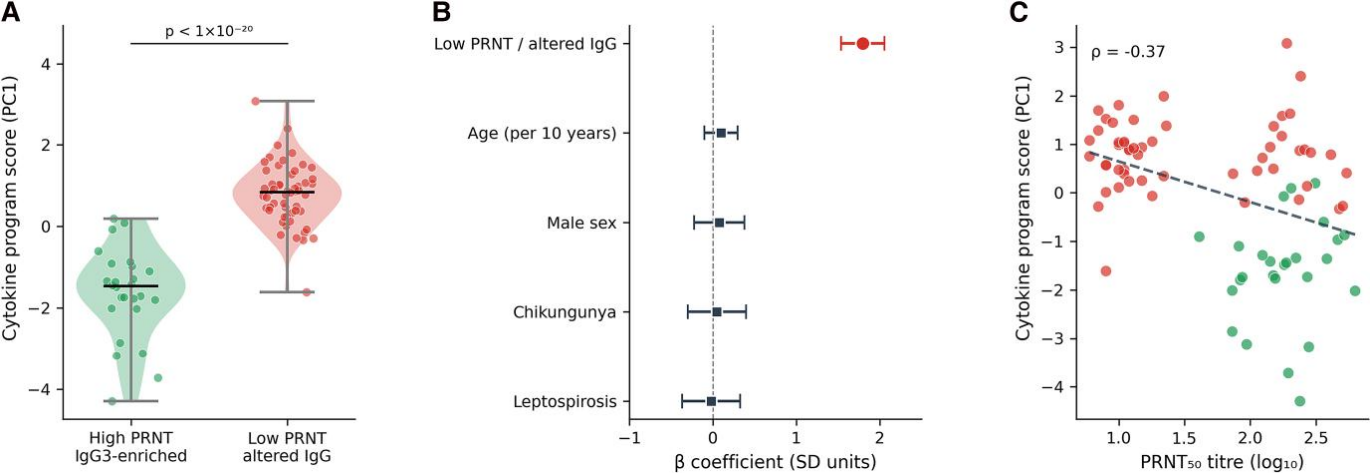
Humoral immune state and cytokine program scores (n = 80). *A*, Violin plots of PC1 scores by humoral immune group. *B*, Forest plot of multivariable regression *β* coefficients. *C*, Scatter plot of PC1 score against log_10_ PRNT_50_ titer (Spearman *ρ* = −0.37). Color indicates humoral immune group (green = high PRNT/IgG3 enriched; red = low PRNT/altered IgG).

Using a multivariable linear regression model, we examined the association between humoral immune group and the immune complex cytokine program score (PC1), adjusting for age, sex, and infection category (Table 5; Figure 5).

**Table 5.** Multivariable Linear Regression of Cytokine Program Score (PC1)

Predictor	*β* (SD Units)	95% CI	*P V*alue
Humoral immune group			
High PRNT/IgG3 enriched	Reference	…	…
Low PRNT/altered IgG	1.80	1.54–2.06	7.18 × 10^−22^
Age (per y)	.01	−.01 to .03	.34
Male sex	.08	−.22 to .38	.60
Infection category			
Dengue	Reference	…	…
Chikungunya	.12	−.25 to .49	.52
Leptospirosis	−.08	−.45 to .29	.67
Model statistics			
Adjusted *R*^2^	.72	…	…

*β* coefficients represent standardized change in PC1 score. Model adjusted for all variables shown.

Abbreviation: CI, confidence interval; IgG, immunoglobulin G; PRNT, plaque reduction neutralization test; SD, standard deviation.

Individuals classified in the low neutralization or altered IgG subclass group exhibited higher inflammatory cytokine program scores compared with those with high neutralization and IgG3-enriched profiles (*β* = +1.80 standard deviation; 95% CI 1.54–2.06; *P* = 7.18 × 10^−22^; Figure 5*A* and 5*B*). This association remained after adjustment for demographic factors and infection category. Age (*β* = .01; 95% CI −.01 to .03; *P* = .34) and sex (*β* = .08; 95% CI −.22 to .38; *P* = .60) were not significantly associated with cytokine program scores.

### Viral Load Does not Confound the Association Between Humoral Immune State and Cytokine Programming

To determine whether circulating viral burden explained the observed association, we examined the relationship between plasma viremia and cytokine program scores in participants with arboviral infection (n = 53). Viremia was not correlated with PC1 score (Spearman *ρ* = −0.15; *P* = .29). In multivariable regression including viremia as a covariate, the association between humoral immune group and PC1 remained essentially unchanged (*β* = 2.98; 95% CI 2.44–3.52; *P* = 7.2 × 10^−15^), while viremia showed no independent association (*β* = .02; *P* = .77). These findings indicate that antibody-dependent immune complex signaling influences cytokine programming independently of viral load (Supplementary Table 2).

### Clinical Context of Immune Complex Cytokine Programming

**Figure 6.**
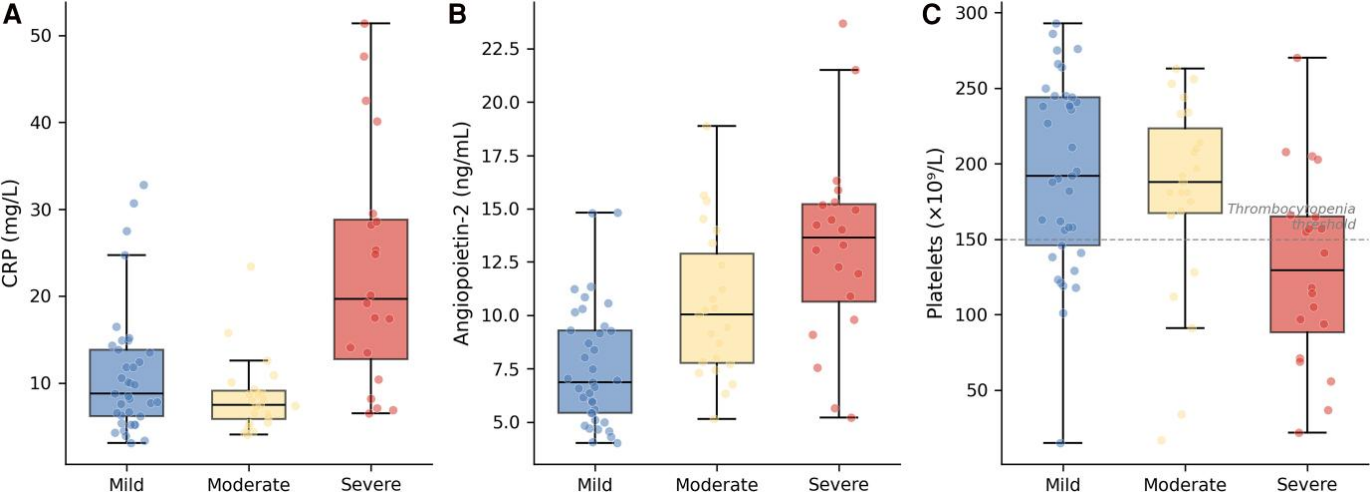
Clinical context of immune complex cytokine programming (n = 80; mild n = 37, moderate n = 23, severe n = 20). *A*, C-reactive protein by disease severity. *B*, Angiopoietin-2 by disease severity. *C*, Platelet counts by disease severity; dashed line indicates thrombocytopenia threshold (150 × 10^9^/L).

To place immune complex cytokine programming in the context of clinical disease, we assessed associations between clinical severity indicators and inflammatory markers (Figure 6). Severe disease was associated with higher C-reactive protein levels, increased angiopoietin-2 concentrations, and lower platelet counts (thrombocytopenia; Figure 6*A*–*C*). These findings suggest that antibody-dependent immune complex cytokine programming is part of a broader inflammatory and endothelial activation environment characteristic of more severe arboviral disease.

## DISCUSSION

In this study, we observed that antibody-dependent immune complex signaling was associated with coordinated inflammatory cytokine responses in patients with acute arboviral infection. Using paired stimulation assays and Fc perturbation experiments, we found that immune complexes were associated with higher pro-inflammatory cytokine production compared with antigen alone and that this difference was partially attenuated by Fc receptor blockade and influenced by antibody subclass composition. Furthermore, we identified a structured cytokine response pattern induced by immune complex stimulation and observed that humoral immune state was strongly associated with this pattern, independent of demographic factors and infection category.

A notable finding of this work is that immune complex stimulation appeared to elicit coordinated cytokine patterns rather than isolated changes in individual cytokines. Principal component analysis revealed a dominant inflammatory axis characterized by positive contributions from IL-6, TNF, IL-1β, and CXCL10 with an inverse contribution from IL-10, consistent with a balance between pro-inflammatory and regulatory signaling. Such coordinated responses have been increasingly recognized as a feature of innate immune activation, reflecting integrated signaling networks rather than linear cytokine cascades [10, 11]. The use of a single, standardized immune-complex stimulus allowed comparison of Fc-dependent inflammatory responses across participants recruited during a heterogeneous outbreak and was designed to isolate host humoral immune state as the primary determinant of immune complex–driven cytokine responses. The use of dengue envelope protein as a standardized stimulus means that the immune complex assay interrogates the preexisting dengue-reactive antibody landscape of each participant rather than pathogen-specific immune complex formation during the acute infection. This design choice was intentional, as it allows isolation of humoral immune state as the principal determinant of cytokine responses, but it should be understood as a measure of Fc-dependent inflammatory potential conditioned on dengue antibody quality rather than as a model of in vivo immune complex biology during the index infection.

The observed differences in cytokine responses under immune complex stimulation conditions are consistent with a role for antibody-dependent signaling in shaping innate immune activation. Immune complexes engage Fcγ receptors on myeloid cells, potentially leading to signal integration with pattern recognition receptor pathways and downstream transcriptional changes [17–19]. The partial attenuation of cytokine responses following Fcγ receptor blockade in our experiments provides evidence that Fc receptor engagement contributes to immune complex–associated inflammatory responses, in line with recent mechanistic studies in viral infection and immune-mediated disease [31–33]. It is important to distinguish the Fc-dependent cytokine signaling pathway interrogated in this study from classical antibody–dependent enhancement of viral entry. Whereas ADE is typically defined as antibody-mediated facilitation of viral infection of Fc receptor–bearing cells, the present work examines a parallel but distinct mechanism: the engagement of Fcγ receptors by antibody–antigen immune complexes leading to downstream inflammatory cytokine production, independent of viral replication. These pathways are not mutually exclusive and may operate concurrently during secondary arboviral infection, but our experimental design was intentionally focused on the signaling rather than the entry component. The Fc perturbation experiments provide convergent but not exhaustive evidence for Fc-dependent signaling. FcγRI (CD64), which binds monomeric IgG with high affinity and may contribute to immune complex uptake, was not blocked. The inhibitory receptor FcγRIIb was not examined, and complement-mediated effects on immune complex processing were not controlled for in the whole-blood format. The modest magnitude of FcγRIIa/IIIa blockade on IL-6 (10% reduction) suggests that these receptors contribute to, but do not fully account for, the immune complex–associated cytokine response and that additional signaling pathways—including complement receptor engagement and FcγRI may operate in parallel.

Our data also suggest that antibody subclass composition may qualitatively influence immune complex cytokine responses. Immunoglobulin G subclasses differ in their affinity for Fcγ receptors and their capacity to induce activating or inhibitory signaling [23–25]. The altered cytokine responses observed following IgG3 depletion suggest that antibody quality, rather than antibody quantity alone, may modulate downstream inflammatory programming. This finding may be relevant in arboviral infections, where secondary infection, cross-reactive antibodies, and evolving humoral immunity are common and have been implicated in immune-mediated pathology [14–16]. An apparent paradox in our data warrants explicit discussion. Immunoglobulin G3 depletion reduced proximal SYK phosphorylation in monocytes (by 54%) yet increased IL-6 output (by 27%). We propose that these observations are reconcilable if IgG3-FcγRIIIa-SYK signaling preferentially drives the regulatory component of the cytokine program specifically IL-10, which loads inversely on PC1 (−0.437) rather than the pro-inflammatory cytokines alone. Under this model, removal of IgG3 interrupts the FcγRIIIa-SYK-IL-10 regulatory circuit, releasing the inflammatory brake and permitting increased IL-6 production through residual FcγRIIa engagement by IgG1 and IgG2 immune complexes. Supporting this interpretation, IgG3 add-back restored pSYK to 93% of intact levels, and this restoration was abolished by FcγRIIIa-specific blockade, confirming the receptor specificity of the IgG3-SYK pathway. We acknowledge that this model remains speculative and would require direct measurement of IL-10 under IgG3-depleted conditions and receptor-specific cytokine dissection experiments for formal validation. The inverse loading of IL-10 on PC1 merits specific comment. Interleukin-10 varied in the opposite direction to IL-6, TNF, IL-1β, and CXCL10 across participants, consistent with its established role as a counter-regulatory mediator induced by overlapping upstream signals but acting through STAT3-dependent pathways to restrain inflammatory gene expression. Because all cytokines were quantified at the same 24-hour time point, this inverse relationship is unlikely to reflect temporal dissociation and more probably represents genuine counter regulation within the immune complex–stimulated response.

A key observation of this study is the strong association between humoral immune state and immune complex cytokine program scores at the population level. Individuals with lower neutralization capacity or altered IgG subclass profiles exhibited substantially higher inflammatory cytokine program scores, even after adjustment for age, sex, and infection category. The magnitude of this association suggests that antibody-dependent immune complex signaling may represent an important axis of immune heterogeneity in acute infection and is consistent with emerging evidence indicating that antibody Fc characteristics, including subclass and glycosylation, may influence innate immune activation and clinical phenotype across infectious diseases [26–28].

Although immune complex cytokine programming occurred within clinically severe inflammatory contexts characterized by endothelial activation, thrombocytopenia, and elevated acute-phase reactants, the ex vivo immune complex signature did not independently predict clinical outcomes in this cohort, and its clinical predictive utility remains to be established. This finding underscores the multifactorial nature of severe arboviral disease, in which endothelial dysfunction, coagulation abnormalities, viral burden, and host metabolic factors interact with immune responses to determine outcome [4–6]. We note that IL-1β, IL-10, and CXCL10 were measured only in the immune complex–stimulated condition, and antigen-only comparisons are therefore available only for IL-6 and TNF. This prevents formal attribution of the full 5-cytokine program to immune complex stimulation specifically. The PCA-derived score should therefore be interpreted as characterizing the coordinated structure of the immune complex cytokine response, rather than as evidence that each constituent cytokine is exclusively immune complex dependent [34].

This study has several limitations that should be considered when interpreting the findings. The mechanistic analyses were conducted in a single regional cohort from southern India during a specific outbreak period, and the sample size of 80 participants, while sufficient for exploratory mechanistic analyses, limits statistical power for fine-grained subgroup comparisons. Replication in independent cohorts from different endemic regions, including populations with distinct arboviral serotype exposure histories and genetic backgrounds, will be necessary to establish the generalizability of these findings. The immunological analyses are cross-sectional, with samples collected at a single time point during acute illness; this design precludes determination of the temporal sequence between humoral immune state and inflammatory cytokine programming, and whether altered antibody subclass composition precedes and drives inflammatory responses, or whether both reflect concurrent processes during acute infection, cannot be resolved without longitudinal sampling. We did not formally classify participants as primary or secondary dengue infections, and the humoral immune grouping used based on PRNT_50_ titer and IgG subclass ratio—serves as a composite proxy for antibody maturity rather than a direct measure of infection history. Future studies incorporating paired acute and convalescent sera would allow formal classification and may reveal additional heterogeneity in immune complex–driven responses between primary and secondary infections. A matched antigen-plus-plasma control condition—using, for example, seronegative donor plasma or heat-inactivated plasma to disrupt immune complex formation while preserving the protein matrix was not included in the present study. While the Fc perturbation experiments provide internal evidence for Fc-dependent signaling, we cannot fully exclude contributions from plasma-derived complement components or nonspecific immunoglobulin effects to the observed cytokine amplification. The ex vivo whole-blood stimulation model cannot recapitulate spatial features of in vivo immune complex biology, including tissue deposition, complement-mediated clearance, endosomal trafficking, and interactions with tissue-resident cells in organ-specific microenvironments. Direct measurement of circulating immune complex concentrations and their subclass composition in plasma would provide complementary in vivo data. Longitudinal studies tracking changes in antibody subclass composition, Fc receptor signaling, and cytokine programming over the course of infection, together with validation in experimental animal models, would be needed to establish a causal role for immune complex signaling in disease progression. Future studies should incorporate such controls and longitudinal designs to strengthen causal attribution and refine these mechanistic links [35–37].

## CONCLUSIONS

This study provides evidence that antibody-dependent immune complex signaling is associated with inflammatory cytokine responses during acute arboviral and leptospiral infection. By integrating functional antibody profiling, Fc perturbation experiments, and multivariable statistical modeling, we describe a potential mechanistic pathway linking humoral immune state to innate inflammatory programming. These findings advance understanding of antibody-mediated immune modulation and suggest that immune complex–Fc receptor signaling may represent a mechanistic pathway worthy of further investigation in clinical cohorts designed to assess its predictive and therapeutic implications. Confirmation in independent populations, together with longitudinal and interventional study designs, will be needed to establish the clinical significance of these observations.

## Supplementary Material

ofag300_Supplementary_Data

## Supplementary Data

Supplementary materials are available at *Open Forum Infectious Diseases* online. Consisting of data provided by the authors to benefit the reader, the posted materials are not copyedited and are the sole responsibility of the authors, so questions or comments should be addressed to the corresponding author.
