# Supplementary material for: Antibody-Dependent Immune Complex Signaling and Inflammatory Cytokine Responses in Acute Febrile Illness: A Mechanistic Study in Arboviral and Leptospiral Infection
Source: Open Forum Infect Dis. 2026 May 14;13(5):ofag300. doi: 10.1093/ofid/ofag300 (PMC13218447; doi:10.1093/ofid/ofag300)
Supplement: ofag300_Supplementary_Data [file ofag300_supplementary_data.zip › Supplementary Table S1 and S2.docx]

**Table S1. Phospho-SYK signalling in CD14+ monocytes following immune complex stimulation (n = 3 donors)**

| **Condition** | **pSYK MFI** | **% of Intact** | **p-value** |
| --- | --- | --- | --- |
| Intact IC | 1281 | 100 | — |
| IgG3-depleted IC | 589 | 46 | 0.005 |
| IgG3-depleted + IgG3 addback | 1185 | 93 | 0.007* |
| IgG3-depleted + IgG1 addback | 862 | 67 | 0.0003† |
| IgG3 addback + FcγRIIIa block | 635 | 50 | 0.007‡ |

*pSYK MFI, phosphorylated SYK median fluorescence intensity; IC, immune complex. *vs IgG3-depleted; †vs IgG3 addback; ‡vs IgG3 addback. p-values from paired t-test.*

**Table S2. Multivariable regression of cytokine programme score (PC1) with viral load adjustment (n = 53 arboviral cases)**

| **Variable** | **β** | **95% CI** | **p-value** |
| --- | --- | --- | --- |
| Low PRNT / altered IgG | 2.98 | 2.44–3.52 | 7.2 × 10⁻¹⁵ |
| Viremia (log₁₀ copies/mL) | 0.02 | −0.13–0.18 | 0.77 |
| Age (per year) | 0.01 | −0.01–0.03 | 0.21 |
| Male sex | −0.16 | −0.61–0.29 | 0.49 |

*Reference group: high PRNT / IgG3-enriched. β coefficients represent standardised change in PC1 score. CI, confidence interval.*
