# Supplementary figures and images for: Antibody-Dependent Immune Complex Signaling and Inflammatory Cytokine Responses in Acute Febrile Illness: A Mechanistic Study in Arboviral and Leptospiral Infection
Source: Open Forum Infect Dis. 2026 May 14;13(5):ofag300. doi: 10.1093/ofid/ofag300 (PMC13218447; doi:10.1093/ofid/ofag300)

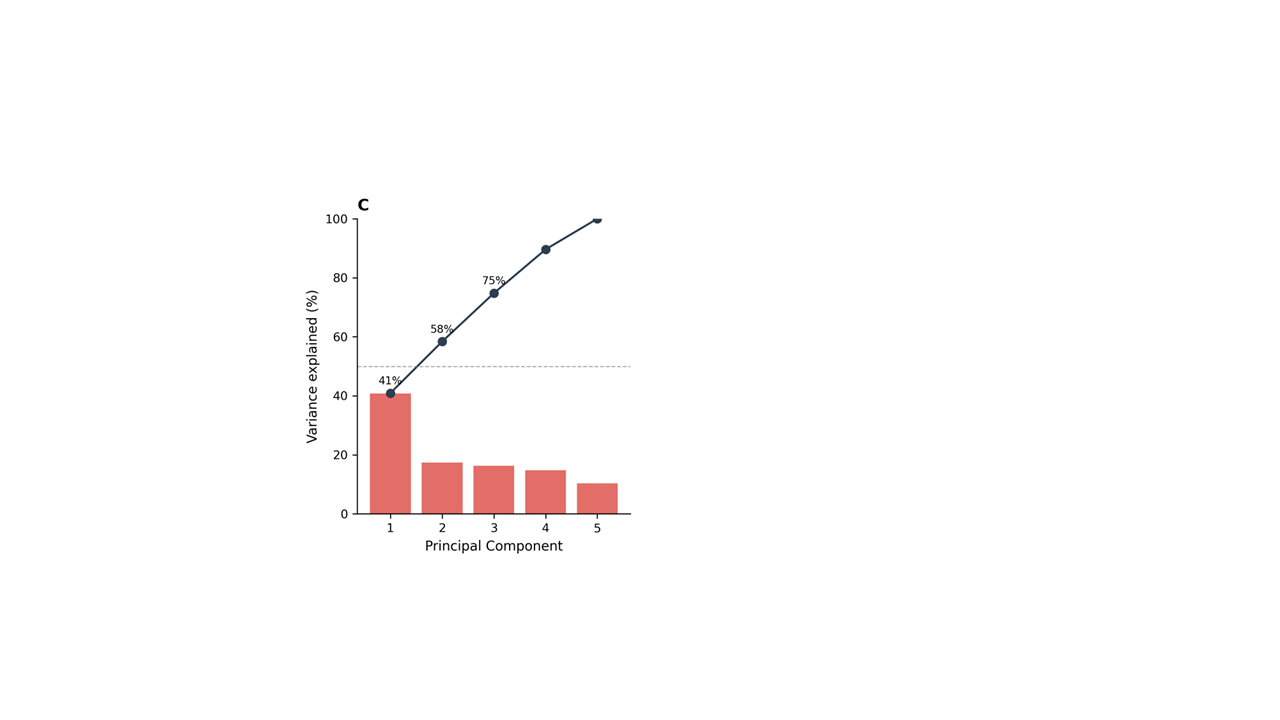

Supplement: ofag300_Supplementary_Data [file ofag300_supplementary_data.zip › Supplementary Figure 1.tiff]
